# Antibacterial properties of natural cinnamon‐alginate fibrous patches produced by modified nozzle‐pressurized spinning

**DOI:** 10.1002/mco2.731

**Published:** 2024-09-10

**Authors:** Yanqi Dai, Merve Gultekinoglu, Cem Bayram, Hettiyahandi Binodh De Silva, Mohan Edirisinghe

**Affiliations:** ^1^ Department of Mechanical Engineering University College London London UK; ^2^ Department of Nanotechnology & Nanomedicine Division Institute for Graduate Studies in Science & Engineering Hacettepe University Ankara Turkey; ^3^ Department of Genetics, Evolution and Environment, The Division of Biosciences University College London London UK

Dear Editor,

Alginate (Alg) is of particular interest as a natural biomaterial due to its unique gelling properties and water absorption capacity. Despite these advantages, the transformation of Alg into commercially value‐added products still faces many challenges.^1^ Our recent study investigates an advanced spinning technology for the facile and large‐scale production of small‐structure Alg antibacterial natural patches incorporated with Ceylon cinnamon.

In this work, nozzle‐pressurized spinning (NPS)^2^ functioned as a jet generation apparatus (Figure S1) spinning Na‐Alg jets into a Ca^2+^‐riched coagulation bath. Ca^2+^ combined with Alg chains in the cross‐linked “egg‐box” model to form Alg fibers. Na‐Alg solutions were generally significantly viscous even at a relatively low concentration (< 5 wt%), exhibiting a pronounced solid‐like behavior. The high pressure applied in NPS effectively mitigates these viscous effects, facilitating Na‐Alg jet formation. Additionally, given the significant production efficiency of NPS, this strategy stands out as a promising approach for the scaling up of Alg fiber production, compared with prevailing methods like electrospinning and wet spinning.

Figure 1A illustrates a marked alteration in the morphology of the obtained Alg products correlating with changes in Na‐Alg/H_2_O solution concentration in NPS. As the concentration increased, the Alg morphology evolved from a thin film to a ribbon‐like structure, ultimately obtaining a filamentous form at the concentration of 3.0 wt%. The rapid increase of solution viscosity with its increasing concentration is a remarkable feature of Na‐Alg/H_2_O solution, accompanied by a significant reduction in its fluidity.^3^ Thereby, the morphology of the resulting Alg products varied. Meeting the critical rheological properties is the key to producing Alg fibers with well‐defined filamentous structures (Figure 1A). Weighing the synergistic effect of solution properties and system parameters, we successfully produced Alg fibers with an average diameter of 10 µm using 3.2 wt% Na‐Alg/H_2_O solution.

**FIGURE 1:**
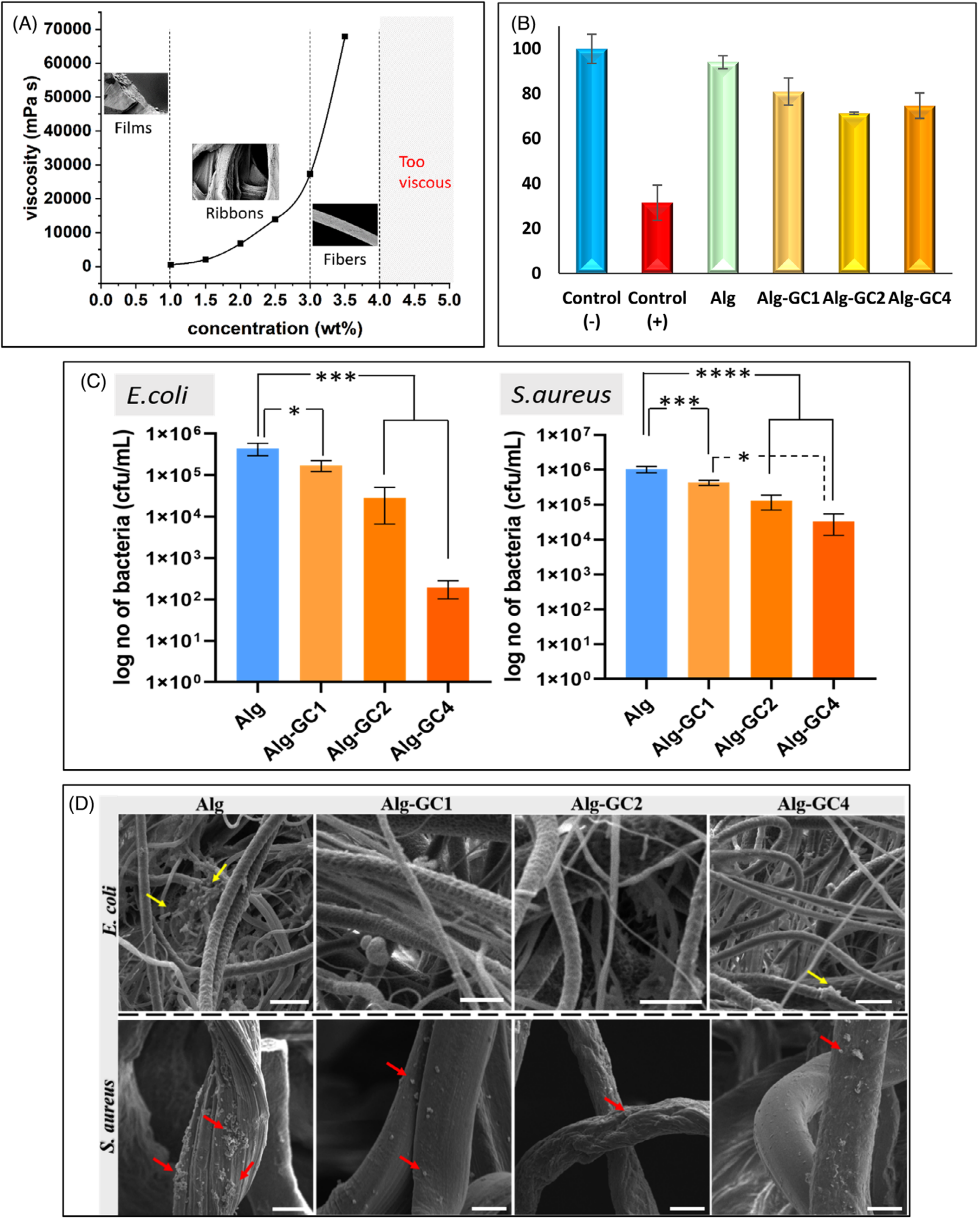
(A) The viscosity profile of sodium alginate solutions and the structures of corresponding alginate products. (Sodium alginate aqueous solutions with a concentration of higher than 4.0 wt% are excessively viscous to be measured). (B) Cell cytotoxicity results of pure alginate (Alg) and Ceylon cinnamon‐alginate fibrous samples with 1%, 2%, and 4% (w/w) of grounded cinnamon (GC), (Alg‐GC1, Alg‐GC2, and Alg‐GC4, respectively). (C) Antibacterial test results of pure alginate (Alg) and Ceylon cinnamon‐alginate fibrous samples with 1%, 2%, and 4% (w/w) of grounded cinnamon (GC), (Alg‐GC1, Alg‐GC2, and Alg‐GC4, respectively) against Gram‐negative *E. coli* (ATCC 25922) and Gram‐positive *S. aureus* (ATCC 29213) bacteria species. (*, ***, and **** indicate the statistically significant difference *p* ˂ 0.05, *p* ˂ 0.005, *p* ˂ 0.0001, respectively). (D) Scanning electron micrographs of fibrous patches with adhered *E. coli* and *S. aureus* bacteria species on pure alginate (Alg) and Ceylon cinnamon‐alginate fibrous samples with 1%, 2%, and 4% (w/w) of grounded cinnamon (GC), (Alg‐GC1, Alg‐GC2, and Alg‐GC4, respectively). Yellow arrows indicate *E. coli* and red arrows indicate *S. aureus* bacteria species. (Scale bar = 10 µm).

Following the established correlation between Alg products and solution properties/processing parameters, Ceylon cinnamon (grounded cinnamon, GC; supplied by HDDES Extracts [PVT] Ltd) was incorporated into Alg fibers using NPS to generate Alg‐GC fibrous patches, with weight ratios of GC of 1%, 2%, and 4% (Alg‐GC1, Alg‐GC2, and, Alg‐GC4). The potential of the resulting Alg‐GC fibrous patches as a biomaterial candidate was evaluated in terms of in‐vitro cell viability and antibacterial properties.

Indirect cytotoxicity tests of pure Alg fibers and Alg‐GC patches were performed by WST‐1 assay according to ISO10993‐5 standard for medical devices.^4^ The results proved that Alg is a biocompatible biomaterial and fibrous Alg and Alg‐GC patches maintain the same cell‐friendly nature (Figure 1B). The pure Alg fibrous patches showed 94 ± 2.8% cell viability compared to the negative control. Positive control that interacted with dimethyl sulfoxide showed 31 ± 7.8% cell viability and it is proven that the L929 cell line is not immortal. Additionally, when GC was added to Alg fibrous patches, no cytotoxicity was observed at any concentration values. The cytotoxicity limit is defined by ISO standard as below 70% cell viability. Alg‐GC1, Alg‐GC2, and Alg‐GC4 sample groups all showed above 70% cell viability compared to the negative control. Thus, it was determined that the Alg, Alg‐GC1, Alg‐GC2, and Alg‐GC4 have cell‐friendly features and significant potential in biomedical applications.

The antibacterial activity of pure Alg and Alg‐GC patches was evaluated to determine their activity to inhibit biofilm formation. *Escherichia coli* (ATCC, #25922) and *Staphylococcus aureus* (ATCC, #29213) bacteria strains were used. Additionally, bacteria adhering to the surface were fixed in place and visualized. All Alg‐GC patches exhibited statistically significant antibacterial activity against the Alg patch for *E. coli*. It was determined that Alg‐GC patches showed antibacterial activity at all concentrations without any cytotoxic response. Alg‐GC sample groups showed less bacterial adhesion than the Alg sample group for both *E. coli* and *S. aureus* bacteria species as is shown in Figure 1C. Alg‐GC2 and Alg‐GC4 sample groups showed log 2 and log 3 decrease in biofilm formation against *E. coli* bacteria, respectively. Additionally, Alg‐GC1, Alg‐GC2, and Alg‐GC4 sample groups exhibited a statistically significant decrease in biofilm formation of *E. coli* compared to the Alg patch (*p ˂ 0.05 and ***p ˂ 0.005). Moreover, statistically significant antibacterial activity was also determined for *S. aureus* bacteria species for all three Alg‐GC patches compared to the Alg patch (*p ˂ 0.05, ***p ˂ 0.005, and ****p ˂ 0.0001). The number of adhered bacteria decreased up to log 3 for GC‐loaded fibrous Alg patches. The antibacterial activity of these fibrous patches was also evaluated by micrographs. Alg, Alg‐GC1, Alg‐GC2, and Alg‐GC4 fibrous patches were subjected to a biological fixation procedure after 24 h incubation with *Gram‐negative E. coli* (ATCC 25922) and *Gram‐positive S. aureus* (ATCC 29213) bacteria species, separately (Figure 1D), which showed similar results with the biofilm formation determination tests. Cinnamon and its derivatives are important antibacterial agent candidates with different strategies that inhibit bacterial cell division, ATPase activity, quorum sensing contact, membrane porins or alter bacterial cell membrane permeability.^5^ The synergistic effect of these features is used to create common solutions to the differences in the membrane structures of *Gram‐negative* and *Gram‐positive* bacteria. According to the antibacterial activity results of *E. coli* and *S. aureus*, Alg‐GC4 showed tremendous potential as an antibacterial wound dressing candidate with high antibacterial activity against both *Gram‐negative* and *Gram‐positive* bacteria species and high cell compatibility.

In summary, the features of precise control and fascinating efficiency of NPS make it a promising strategy to scale up Alg fiber patch manufacturing with low‐cost and facile behavior. Ceylon cinnamon was loaded into these Alg fiber structures. The antibacterial test results showed that these natural patches exhibited a notable inhibitory effect on bacterial growth, with the antibacterial efficacy demonstrating a distinct dose dependence against both *Gram‐positive* and *Gram‐negative* bacteria species. It was determined that increasing cinnamon concentrations increased the antibacterial activity. Additionally, the cytotoxicity test results exhibited significant cell activity of Alg and cinnamon‐Alg fibrous patches, highlighting its potential as a sustainable biocompatible biomaterial.

## AUTHOR CONTRIBUTIONS

*Yanqi Dai, Merve Gultekinoglu, Cem Bayram, and Hettiyahandi Binodh De Silva*: performed experiments. *Yanqi Dai and Merve Gultekinoglu*: analyzed data and wrote and revised the manuscript. *Mohan Edirisinghe*: conceived and supervised the project. All authors have read and approved the final manuscript.

## CONFLICT OF INTEREST STATEMENT

HDDES Extracts (Pvt) Ltd partly provides financial support to this study. Mohan Edirisinghe is an Editorial board member of MedComm. Mohan Edirisinghe was not involved in the journal's review or decisions related to this manuscript. The other authors declare no conflict of interest.

## FUNDING INFORMATION

China Scholarship Council, Engineering and Physical Sciences Research Council (EP/S016872/1, EP/N034228/1, EP/L023059/1) and HDDES Extracts (Pvt) Ltd

## ETHICS STATEMENT

Not applicable

## Supporting information

Supporting Information

## Data Availability

The authors confirm that the data supporting the findings of this study are available within the article and its Supporting Information. These data can be accessed by contacting the corresponding author, upon reasonable request.

## References

[1] Kumar S , Malviya R , Sundram S . Management of peripheral nerve injuries using natural based biomaterials and their derivatives: advances and prospective. MedComm Biomater Appl. 2024;3(1):e72.

[2] Dai Y , Ahmed J , Delbusso A , Edirisinghe M . Nozzle‐pressurized gyration: a novel fiber manufacturing process. Macromol Mater Eng. 2022;307(9):2200268.

[3] Brzezińska M , Szparaga G . The effect of sodium alginate concentration on the rheological parameters of spinning solutions. Autex Res J. 2015;15(2):123‐126.

[4] Aksoy EA , Taskor G , Gultekinoglu M , Kara F , Ulubayram K . Synthesis of biodegradable polyurethanes chain‐extended with (2S)‐bis(2‐hydroxypropyl) 2‐aminopentane dioate. J Appl Polym Sci. 2018;135(5):45764.

[5] Vasconcelos NG , Croda J , Simionatto S . Antibacterial mechanisms of cinnamon and its constituents: a review. Microb Pathog. 2018;120:198‐203. 29702210 10.1016/j.micpath.2018.04.036

