# Supplementary material for: Antibacterial properties of natural cinnamon‐alginate fibrous patches produced by modified nozzle‐pressurized spinning
Source: MedComm (2020). 2024 Sep 10;5(9):e731. doi: 10.1002/mco2.731 (PMC11387719; doi:10.1002/mco2.731)
Supplement: Supplementary file 1 — Supporting Information [file MCO2-5-e731-s001.docx]

**Antibacterial properties of natural cinnamon-alginate fibrous patches produced by modified nozzle-pressurized spinning**

Yanqi Dai^1^, Merve Gultekinoglu^2^, Cem Bayram^2^, Hettiyahandi Binodh De Silva^3^, Mohan Edirisinghe^1,*^

^1^Department of Mechanical Engineering, University College London, London, UK

^2^Department of Nanotechnology & Nanomedicine Division, Institute for Graduate Studies in Science & Engineering Hacettepe University, Ankara, Turkey

^3^Department of Genetics, Evolution and Environment, The Division of Biosciences, University College London, London, UK

∗Correspondence

Mohan Edirisinghe, Department of Mechanical Engineering, University College London, London, WC1E 7JE, UK.

E-mail: [m.edirisinghe@ucl.ac.uk](mailto:m.edirisinghe@ucl.ac.uk)

**Materials and Methods**

1. Preparation of pure Alg and cinnamon-Alg samples

Na-Alg/H_2_O solutions with a series of concentrations ranging from 1.0 to 3.5 wt% were prepared by dissolving specific amounts of Na-Alg powders in distilled water and mechanically stirring for 48 h. 3.2 wt% Na-Alg solutions incorporated with ground cinnamon (GC) of weight ratios to the polymer of 1%, 2%, and 4% (w/w) were prepared following the same procedure.

After the preliminary experiment, the prepared solutions were subjected to NPS (Figure S1) with rotational speed of 11 000 rpm, working pressure of 2 × 10^5^ Pa, and air gap length of 6 mm. A 3.5 wt% CaCl_2_ bath was used as the coagulant. The resulting Alg gels after NPS were collected from the coagulant, preliminarily dried with absorbent tissue, and then oven-dried at 55°C until completely dry.

1. Scanning electron microscope (SEM) studies

The morphology of the resulting Alg and Alg-GC samples was examined using scanning electron microscopy (GeminiSEM 360, ZEISS, Germany). Observations were conducted at an acceleration voltage of 1 kV for the SEM.

1. *In-vitro* Cytotoxicity testing

The cytotoxicity tests of pure Alg and Alg-GC fibrous samples were performed according to the ISO10993-5 standard. L929 mouse fibroblast cell line ATCC-NCTC clone 929:CCL1 was used. All the samples were sterilized prior to the test. The test samples were prepared according to ISO standards with the 6 cm^2^/mL sample-medium ratio. The Alg-GC samples were tested by WST-1 Assay (Cayman, #600485) to examine their cytotoxic response. The test samples were immersed in cell culture medium and kept at 37 °C for 72 h. The cell culture medium was composed of 90% DMEM (Dulbecco's Minimum Essential Medium), 10% FBS (Fetal Bovine Serum), 2 mM L-glutamine, and 100 IU/mL penicillin/streptomycin. The cells were seeded in a 96-well plate at 1×10^4^ cells/well. Then the incubated cell culture medium was interacted with cells for 24 h. After 24 h, 10 µL WST-1 assay reagent was added to each well, and cells were incubated for 120 min. The 96-well plate (n=3) was measured at 450 nm absorbance by ELISA plate reader (SpectroStar nano, BMG Labtech). Non-reacted cell culture medium was used as negative control and the 10% DMSO-90% medium was used as positive control.

1. Antibacterial testing

The antibacterial activity of pure Alg and Alg-GC fibrous samples was evaluated using *Escherichia coli* (*E. coli*, ATCC, #25922) and *Staphylococcus aureus* (*S. aureus*, ATCC, #29213) bacteria strains. The bacterial solution (Luria-Bertani medium, Sigma, #L7275) was adjusted to 0.4 OD. The fibrous samples were placed into a 48-well plate and 0.5 mL bacteria suspension was added (n=3). The samples were incubated for 24 h at 37°C in a shaker incubator. After 24 h, samples were collected and kept at 1 mL PBS (Sigma Aldrich, #P4417) solution. Then the test samples were ultrasonicated for 15 min. The collected PBS solution was serially diluted and 10 µL samples were cultivated to agar plates (n=3). After 24 h, colony-forming units were counted and the antibacterial activity-antibiofilm formation was calculated. Additionally, after 24 hours of incubation, the bacteria adhering to the surface were fixed in place and visualized by SEM. In the fixation procedure, the first cross-linking was performed with glutaraldehyde for 30 mins. Then, incubation was carried out with serial alcohol solutions (50, 60, 70, 80, 90, 95, and 100%) for 15-minute periods to gradually remove the water from the samples. All samples were gold-plated prior to SEM imaging.

**Supplementary Figure**





**Figure S1** Schematic diagram of a modified nozzle-pressurized spinning (NPS) setup incorporated with a CaCl_2_ coagulation bath. Red arrows indicate the resulting Na-Alg jets by NPS.
